# Cross-resistance in *Alternaria brassicicola* from naturally infested broccoli seeds against two succinate dehydrogenase inhibitor fungicides

**DOI:** 10.1128/aem.01083-25

**Published:** 2025-09-04

**Authors:** Navjot Kaur, Anoop A. Malik, Daniel G. Cerritos-Garcia, Rachel A. Koch Bach, Sydney Everhart, Bhabesh Dutta

**Affiliations:** 1 Department of Plant Pathology, University of Georgia 1355, Tifton, Georgia, USA; 2 Department of Plant Science and Landscape Architecture, University of Connecticut 7712, Storrs, Connecticut, USA; The University of Tennessee Knoxville, Knoxville, Tennessee, USA

**Keywords:** naturally infested commercial broccoli seeds, cross-resistance, succinate dehydrogenase inhibitor (SDHI) fungicides, mutation, *Alternaria brassicicola*

## Abstract

Seeds can serve as a vehicle for the dissemination of pests and pathogens around the world. We recently demonstrated the association of pathogenic *Alternaria brassicicola* isolates with reduced sensitivity to azoxystrobin (quinone-outside inhibitor [QoI]) in naturally infested commercial broccoli seeds. In this study, we further demonstrate that these isolates were also resistant to two succinate dehydrogenase inhibitor (SDHI) fungicides. Sensitivity of representative *A. brassicicola* isolates (*n* = 58) from naturally infested broccoli seedlots to QoI and SDHI fungicides was evaluated under *in vitro* conditions. Interestingly, 15% (*n* = 9/58) of the *A. brassicicola* isolates with reduced sensitivity to azoxystrobin also displayed reduced sensitivity to two commonly used SDHI fungicides (boscalid and penthiopyrad) in broccoli, indicating a potential case of cross-resistance to SDHI fungicides. Ninety-three percent of the isolates (*n* = 54/58) were resistant to both boscalid and penthiopyrad, while 100% isolates displayed sensitivity to fluopyram. Sequence analysis of *sdh* genes revealed the presence of only one point mutation (H134R) in the *sdh*C gene in isolates that displayed resistance to boscalid and penthiopyrad. We also developed and validated allele-specific primers targeting the H134R mutation for rapid screening of SDHI resistance in *A. brassicicola*. We also found that boscalid-resistant isolates exhibited significantly reduced mycelial growth. However, spore germination rates among different resistant isolates were not different, suggesting that resistant isolates remain competitive in natural populations. Overall, this study provides the first evidence of fungicide resistance to SDHI fungicides in *A. brassicicola* isolated from naturally infested broccoli seeds and underscores the importance of seeds as a potential source for introducing fungicide resistance across geographical locations.

**IMPORTANCE:**

*Alternaria brassicicola* is a fungal seed-borne pathogen that can be disseminated via commercial seeds across transplant houses and commercial broccoli fields. Our study provides the first evidence that commercial broccoli seeds can harbor pathogenic *A. brassicicola* isolates with cross-resistance to two succinate dehydrogenase inhibitor (SDHI) fungicides. We observed that 93% of the *A. brassicicola* isolates from naturally infested commercial broccoli seeds contained a point mutation that conferred resistance to two SDHI fungicides (boscalid and penthiopyrad). Furthermore, we developed a PCR-based allele-specific assay for the rapid detection and monitoring of fungicide resistance. Our study highlights the importance of seed health testing and potential dissemination of fungicide-resistant isolates locally and globally, thus impacting disease management strategies.

## INTRODUCTION

Seeds can serve as a vehicle for the dissemination of pests and pathogens around the world (1). Seeds can act as a carrier and may aid in disseminating and establishing pathogens in different geographic locations, leading to initiation and upsurge in disease epidemics (2, 3). Thus, it is of paramount importance to understand the mechanisms of natural infestation of seeds by plant pathogens and assess if pathogens associated with infested seeds are sensitive to commonly used fungicides that are employed in disease management programs. *Alternaria brassicicola* (Schwein.) is an important seedborne pathogen of brassica crops in the United States. It is a necrotrophic fungal pathogen that causes Alternaria leaf blight and head rot (ABHR) in broccoli (4–7). Recently, it was documented that *A. brassicicola* is the predominant species along with *A. japonica* in the eastern United States, and these fungal species were implicated with increased emergence and outbreaks of ABHR epidemics (8, 9). An important but underexplored route of pathogen introduction is via naturally infested broccoli seeds. While there are a few reports of natural seed infestation of *A. brassicicola* in brassica crops around the world (10–12), natural *A. brassicicola*-infestation in commercial broccoli seeds was recently documented in the United States (13). In our previous study, we provided evidence for the potential presence of pathogenic and aggressive *A. brassicicola* isolates with reduced sensitivity to azoxystrobin in naturally infested commercial broccoli seedlots (13). A similar report of seed-borne inoculum with fungicide-resistant pathogen was reported in *Didymella bryoniae* (currently known as *Stagonosporopsis citrulli*), a causal agent of gummy stem blight in watermelon (14). The authors recovered only three isolates while screening 5,800 commercial watermelon seeds. Apparently, these isolates exhibited resistance to thiophanate-methyl but were sensitive to boscalid, azoxystrobin, and tebuconazole (14). In our case, apart from azoxystrobin (13), the seedborne *A. brassicicola* isolates were not investigated for their sensitivity to succinate dehydrogenase inhibitor (SDHI) fungicides.

Since *A. brassicicola* can be seed-borne and seed transmitted, we hypothesized that naturally infested commercial broccoli seeds could harbor *A. brassicicola* isolates that are resistant to commonly used SDHI fungicides. The occurrence of less SDHI-sensitive *A. brassicicola* populations could potentially impact fungicide programs commonly utilized to manage ABHR. Hence, the objectives of this study were to characterize *A. brassicicola* isolates from naturally infested commercial broccoli seedlots for their sensitivity to three SDHI fungicides (boscalid, penthiopyrad, and fluopyram), and identify mutations in *sdh* genes conferring SDHI fungicide resistance. Furthermore, we also developed allele-specific PCR primers for rapid screening of SDHI resistance corresponding to the *sdhC* gene and analyzed the phylogenetic and evolutionary relationships of SDHI genes among different *Alternaria* species. In addition, we also determined the fitness parameters in terms of mycelial growth and spore germination of *A. brassicicola* isolates under *in vitro* conditions. Moreover, by integrating sequence-based phylogeny with exon-intron structural analysis, our study aimed to uncover whether resistant isolates of *A. brassicicola* share a close evolutionary relationship with other *Alternaria* spp. harboring similar mutations, thus enhancing our understanding of SDHI resistance dynamics.

## MATERIALS AND METHODS

### *Alternaria brassicicola* isolates

In our previous study, we collected a total of 133 isolates from naturally infested commercial broccoli seedlots from two commonly grown cultivars (Cultivar 1 [EC] and Cultivar 2 [ED]) in the Eastern US (13). The isolates were further identified as *A. brassicicola* using a multi-gene phylogeny and species-specific PCR assay (14, 15). Pathogenicity was evaluated under *in vitro* conditions using a seedling grow-out assay (13). A majority of the *A. brassicicola* isolates were pathogenic and highly aggressive on broccoli foliage. Fifty-eight representative *A. brassicicola* isolates (one from each infected seedlot) were selected for screening sensitivity to SDHI fungicides.

### Fungicide sensitivity of *A. brassicicola* isolates to three SDHI fungicides

A radial growth assay was used to determine the sensitivity of *A. brassicicola* to three SDHI fungicides that includes boscalid, fluopyram, and penthiopyrad, as described previously (15–17). Full-strength potato dextrose agar (PDA) was amended with various concentrations of three fungicides. The concentrations used for boscalid were 0.01 parts per million (ppm), 0.1 ppm, 1 ppm, 10 ppm, and 50 ppm, along with a control (non-amended with fungicide), while for penthiopyrad and fluopyram, the following concentrations were used: 0.01 ppm, 0.1 ppm, 1 ppm, 10 ppm, and a control. Stock solutions of 50,000 mg/mL (100% a.i.; Sigma-Aldrich) for boscalid were prepared by dissolving 50 mg of fungicide into 1 mL of acetone. While a stock solution of 10,000 mg/mL (100% a.i.; Sigma-Aldrich) for fluopyram and penthiopyrad was prepared by dissolving 10 mg of each fungicide into 1 mL of acetone. Fungicide concentrations of 0.01, 0.1, 1, 10, and 50 ppm were prepared by serial dilution, and 1 mL of fungicide solution was added per liter of PDA after cooling the media to 55°C. The concentration of acetone was maintained at 0.1% vol/vol in the non-amended medium (the same concentration as the fungicide-amended media). Isolates were grown on full-strength PDA plates with 12 h of light and 12 h of dark for 7–8 days at 24°C. A 7 mm agar plug from a 7-day-old grown *A. brassicicola* isolates was used for the fungicide sensitivity assay. The fungicide-amended plates, along with control, were incubated at 24°C in the dark for 7 days, and colony diameter was recorded in three perpendicular directions. Three replicates were used per fungicide concentration in a completely randomized design, and the experiment was conducted twice independently. The fungicide concentration that reduced mycelial growth by 50% relative to the control (effective concentration or EC_50_) in parts per million (ppm) was determined for each isolate following the procedure of Reimann and Deising (18). Briefly, the regression line between the logit-transformed fungicide efficiency and the log-transformed fungicide concentrations was calculated using linear regression in Microsoft Excel, and the mean EC_50_ was determined for each isolate. The resistance factor (RF) was calculated by dividing the effective fungicide concentration (EC_50_) value of each isolate by the EC_50_ value of the sensitive isolate. No EC_50_ was calculated for boscalid and penthiopyrad as the isolates grew at the highest concentration screened, which were 50 ppm and 10 ppm, respectively. For fluopyram, an analysis of variance (ANOVA) was conducted in R (v 4.3.1) to determine if there was a significant difference in EC_50_ values among isolates. Tukey’s honest significant difference (HSD) test was used as post hoc test to determine pairwise significant differences.

### Primer design and validation for SDHI gene subunits

To assess whether there is genetic variability and identify mutations in SDHI genes within *A. brassicicola*, we generated high-throughput sequencing data for four previously purified isolates. Details of these isolates, including collection information and host, are presented in Table S2. Briefly, high-quality genomic DNA was extracted from spores and mycelia using the Qiagen DNeasy Plant Maxi Kit (Hilden, Germany) following the manufacturer’s protocol. Genomic DNA was submitted for high-throughput sequencing to the Center for Genome Innovation at the University of Connecticut, where 100 bp paired-end reads were sequenced on an Illumina NovaSeq 6000. Raw reads were trimmed and filtered for quality using Trimmomatic (19). The trimmed reads were then assembled *de novo* using SPAdes v 3.15.3 (20) with the default parameters, and scaffolds less than 1,000 bp were discarded.

Primer design and sequence analyses for the SDHI gene subunits were conducted using Geneious Prime version 2024.0.7 (Biomatters Ltd., Auckland, New Zealand). FASTA files containing all contigs from the genomes of the four *A. brassicicola* isolates were imported into the software. To identify SDHI genes from the *A. brassicicola* genomes, annotated sequences from *A. solani* and *A. alternata* for subunits *sdh*B, *sdh*C, and *sdh*D were retrieved from the NCBI database (accession numbers for sequences are listed in Table S3) (16, 21, 22). Subunit sequences were aligned to the *A. brassicicola* genomes using the MegaBLAST algorithm with a 200 bp overhang at each end to account for flanking regions. Results showed all SDHI subunits from *A. solani* and *A. alternata* mapped to the same sites within each *A. brassicicola* genome, which were analyzed for sequence variation using the *De Novo* assembly tool in Geneious Prime. The analysis showed that these SDHI subunits across the multiple *A. brassicicola* isolates were monomorphic.

Primers for each putative SDHI subunit were designed using Primer3 (v.2.3.7), employing default parameters (Table 1). Two primer sets were designed to tile the >1,000 bp *sdh*B subunit, whereas *sdh*C and *sdh*D subunits required one primer set per subunit. Validation through PCR amplification and Sanger sequencing was performed using four *A. brassicicola* isolates identified as follows: 19-5 (sensitive to QoI; Georgia, USA), EC43-R2-2 (sensitive to Boscalid; Georgia, USA), ED63-R3-1 (resistant to Boscalid; Georgia, USA), and 23209 (resistant to Boscalid; New York, USA). Reactions were prepared in 20 µL volumes using BIONEER PCR premix tubes. Each reaction had 2 µl DNA (~20 ng), 1 µL forward primer (10 µM), 1 µL reverse primer (10 µM), and 16 µL nuclease-free water. Amplicon sizes were confirmed by gel electrophoresis prior to sequencing (Eurofins Genomics, Louisville, KY, USA). Gene sequencing results confirmed the successful amplification of the target regions for all isolates. For the *sdh*B gene, sequence similarities of isolates to the reference were all close to 100% (19-5: 100%, 23209: 99.9%, EC43-R2-2: 100%, ED63-R3-1: 100%). For the *sdh*C gene, sequence similarities of isolates to reference ranged from 98.7 to 99.1% (19-5: 99.0%, 23209: 98.7%, EC43-R2-2: 99.1%, ED63-R3-1: 98.7%). For the *sdh*D gene, sequence similarities of isolates to reference ranged from 98.7 to 99.3% (19-5: 99.1%, 23209: 99.0%, EC43-R2-2: 99.3%, ED63-R3-1: 98.7%). Query coverage was 100% for all isolates and *sdh* genes. To confirm primers were amplifying the target regions, sequences from each isolate were aligned to different reference sequences of each subunit. The sequences from the different accessions from *A. alternata* and *A. solani* (Table 1), as well as a consensus sequence of the subunits of the *A. brassicicola* genomes, were used as reference sequences. Sequences were first trimmed using the *Trim ends* option, and the default 0.05 error probability was selected. After trimming, the *Align/Assemble* option was selected, and then the *Map to reference* option was used to assemble sequences. The default standard Geneious assembler was used to align sequences to the respective references. All the sequences from isolates 19-5, 18062, F6A2, and F6A9 from each subunit were successfully assembled to the respective reference sequences.

**TABLE 1:** Primers developed for molecular detection of succinate dehydrogenase-inhibiting (SDHI) fungicide-resistant *Alternaria brassicicola* isolates

SDHI subunit	Primer name	Forward (5′−3′)	Reverse (5′−3′)	Expected amplicon size (bp)
*sdh*B^*a*^	AbsdhB1	TTCGTAGCAAGTGTTGACGTTTT	GAAGAGCGTCATGTCCGGC	850
*sdh*B	AbsdhB2	TCGCGAAGGTATCTGCGGAA	GGAAAGGTGAGGTTCACTGACT	845
*sdh*C	AbsdhC	GACATGTGCGTGGCTCCAA	TGTCCGCAAAGTGGATGACT	963
*sdh*D	AbsdhD	GCGACTAGGCTATCTTGAGTATGA	CAAATCTGCGATCACGGGAT	977

^*a*^ Two primer pairs for the sdhB subunit were designed since the amplicon size was more than 1,000 bp.

### Allele-specific validation and primer development for mutation in the *sdh*C gene

The mutation H134R identified in the *sdh*C gene was investigated by designing allele-specific primers to validate its presence among *A. brassicicola* isolates. This mutation was identified in *A. brassicicola* isolates (*n* = 54) that showed resistance to SDHI fungicides (boscalid and penthiopyrad) in the fungicide sensitivity assay, while the remaining four sensitive isolates lacked the mutation. The mutation was confirmed through the MUSCLE multiple sequence alignment tool in Geneious Prime (v.2024.0.7) using default parameters. Alignment results demonstrated high conservation of the mutation site among resistant isolates.

Allele-specific primers were designed using Primer3 software. The design parameters were set to maximize specificity at the 3′ end of the primers to distinguish the mutant allele (H134R) from the wild-type sequence. Two primer sets were created: one specific to the mutant allele and the other to the wild-type sequence. The primer sequences are listed in Table 2.

**TABLE 2:** Allele-specific primers for validation of the H134R mutation in *Alternaria brassicicola* isolates that displayed resistance to three SDHI fungicides (boscalid, penthiopyrad, and fluopyram)

Prime name	Sequence (5′−3′)	Specificity	Expected amplicon size (bp)
sdhC_H134R_F	CGCTTTCCCCTTCTTCTTCCG	Mutant allele	326
sdhC_WT_F	CGCTTTCCCCTTCTTCTTCCA	Wild-type allele	326
sdhC_Reverse	GACATGTGCGTGGCTCCAA	Common reverse	

PCR validation of the mutation was performed using BIONEER PCR premix tubes in 20 µL reaction volumes. Each reaction contained 2 µl DNA (~20 ng), 1 µL forward allele-specific primer (10 µM), 1 µL reverse primer (10 µM), and 16 µL nuclease-free water. PCR conditions included an initial denaturation at 94°C for 2 minutes, followed by 35 cycles of 94°C for 30 seconds, 63°C for 30 seconds, and 72°C for 60 seconds, with a final extension at 72°C for 10 min. PCR products were analyzed via gel electrophoresis on a 1.5% agarose gel stained with Gelgreen Nucleic Acid Gel Stain (Biotium).

### Phylogenetic and genomic analyses of SDHI genes

The evolutionary relationships and genomic structures of the SDHI genes were analyzed using full-length sequences of *sdh*B, *sdh*C, and *sdh*D subunits. The full-length sequences of these three subunits were retrieved from the NCBI database for four species of *Alternaria: A. solani, A. alternata, A. tenuissima,* and *A. arborescens*. These sequences, along with the corresponding SDHI gene sequences from *Alternaria brassicicola* and an outgroup sequence of *Stemphylium vesicarium*, were utilized to construct phylogenetic trees for each subunit. Phylogenetic analysis was performed by MEGA11 using the maximum likelihood method with 500 bootstrap replicates to ensure the robustness of the inferred evolutionary relationships. This approach allowed for a detailed comparison of the SDHI genes across species, highlighting evolutionary divergence or conservation patterns.

In addition, the exon-intron arrangements of the *sdh*B, *sdh*C, and *sdh*D genes were examined for all *Alternaria* species. The genomic structures were analyzed to determine the specific organization of coding (exons) and non-coding (introns) regions in each species. This structural analysis was carried out to explore potential differences or similarities in the arrangement of these regions, which could provide insights into functional conservation, gene regulation, or evolutionary adaptations. The combination of phylogenetic relationships with exon-intron structural comparisons offers a comprehensive understanding of the SDHI gene architecture and evolution across closely related fungal species.

### Pathogen fitness test

The pathogen fitness characteristics were determined by evaluating mycelial growth and spore germination *in vitro* as described earlier (23, 24). Briefly, a representative set of 12 isolates from each fungicide-resistant or -sensitive category was used to determine the pathogen fitness. After the EC_50_ value determinations from the fungicide sensitivity study, a set of isolates with varying sensitivity profiles to azoxystrobin and boscalid was selected for the pathogen fitness assay; set 1: isolates resistant to boscalid (Bos^R^, *n* = 4); set 2: isolates that are sensitive to both boscalid and azoxystrobin (Bos^S^ +Azoxy^S^, *n* = 4); and set 3: isolates that are resistant to boscalid and less sensitive to azoxystrobin (Bos^R^ +Azoxy^R^, *n* = 4). Isolates were grown on full-strength PDA and were incubated under 12 h of light and 12 h of darkness at 25°C for 7 days. After 7 days of growth, four 7 mm mycelial plugs per isolate were cut from the margins of actively growing cultures and transferred to the center of fresh PDA plates. Plates were incubated in the dark at 25°C for 5 days. Radial growth was recorded first at the widest radial growth and perpendicular to that using a ruler. Four replicates per isolate were used in a completely randomized design, and the experiment was repeated independently. The mean of two measurements was calculated, and the mycelial growth area (mm^2^) was determined. ANOVA was conducted in R (v 4.3.1) to determine whether there was a difference in mycelial growth among isolates. Tukey’s honest significant difference (HSD) test was used *post hoc* to determine significant pairwise differences.

For the spore germination assay, the same set of isolates (*n* = 12) from three categories or sets as described above were used in the *in vitro* study. Isolates were grown as described above in the mycelial growth assay. Spore suspensions were prepared in distilled sterile water from 7-day-old cultures, and the conidial concentration for each isolate was adjusted to 1 × 10^5^ spores/mL using a hemocytometer. Then, for each isolate, 100 µL aliquots of the spore suspensions were spread onto the surface of water agar (2% wt/vol) plates. The plates were incubated in the dark at 25°C for 24 h. One hundred arbitrarily selected spores were counted, and conidia were considered germinated if the size of the germ tube was greater than the length of the conidium. Four replicates per isolate were used in a completely randomized design, and the experiment was repeated independently. ANOVA was determined for the percent germination of conidia in R (R version 4.3.1). Tukey’s honest significant difference (HSD) test was used *post hoc* to determine significant pairwise differences.

## RESULTS

### Sensitivity of *A. brassicicola* isolates from naturally infested commercial broccoli seeds to three SDHI fungicides

**Fig 1:**
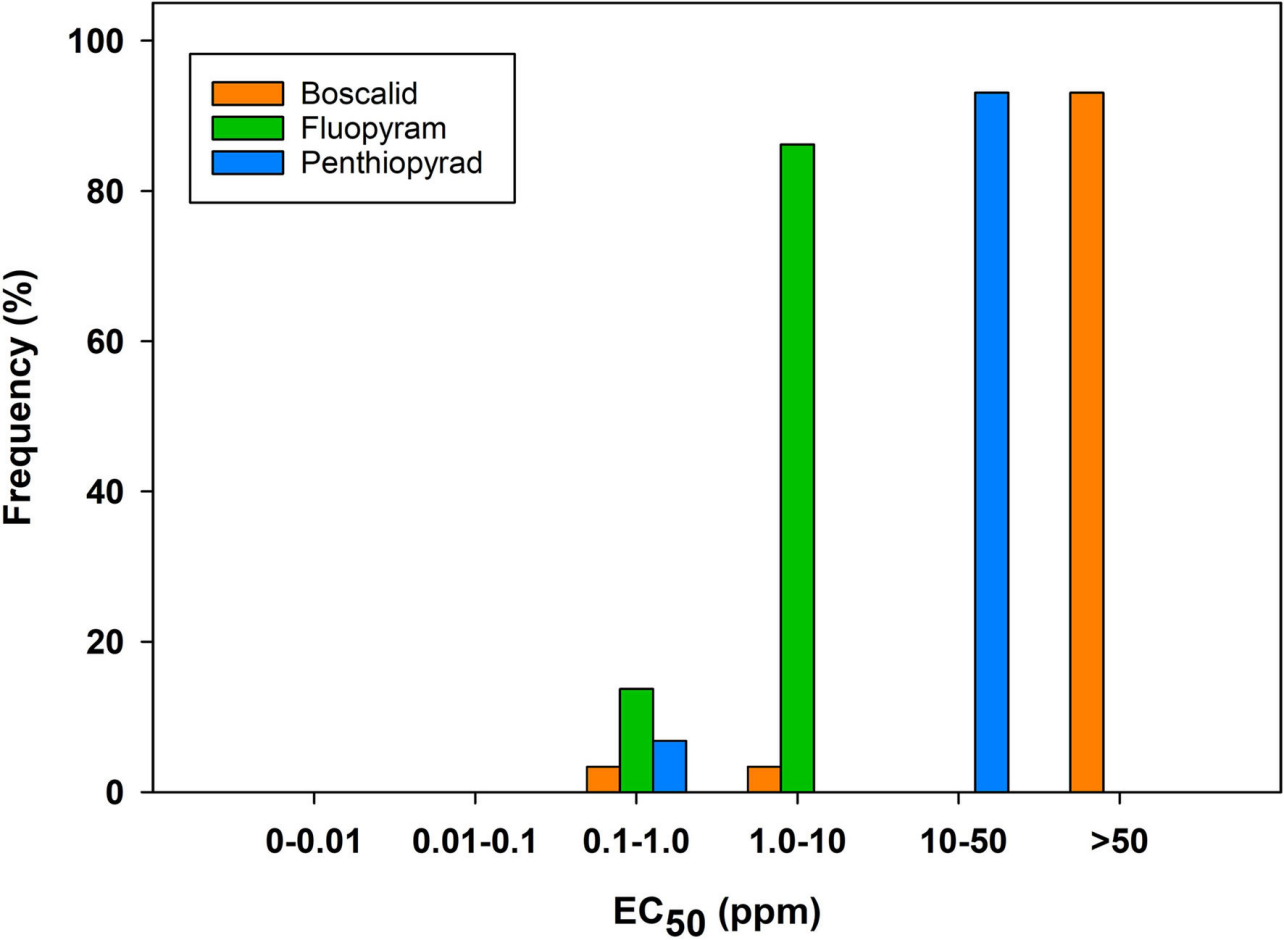
*In vitro* fungicide sensitivity profiles of *Alternaria brassicicola* isolates (*n* = 58) from naturally infested commercial broccoli seedlots to three succinate dehydrogenase inhibitor fungicides: boscalid, penthiopyrad, and fluopyram. Values on the *x*-axis are a range of EC_50_ values (effective fungicide concentration [in parts per million] at which the growth of an isolate is reduced by 50% compared with control) conducted using a radial growth assay. The *y*-axis indicates the frequency of isolates in each category.

**Fig 2:**
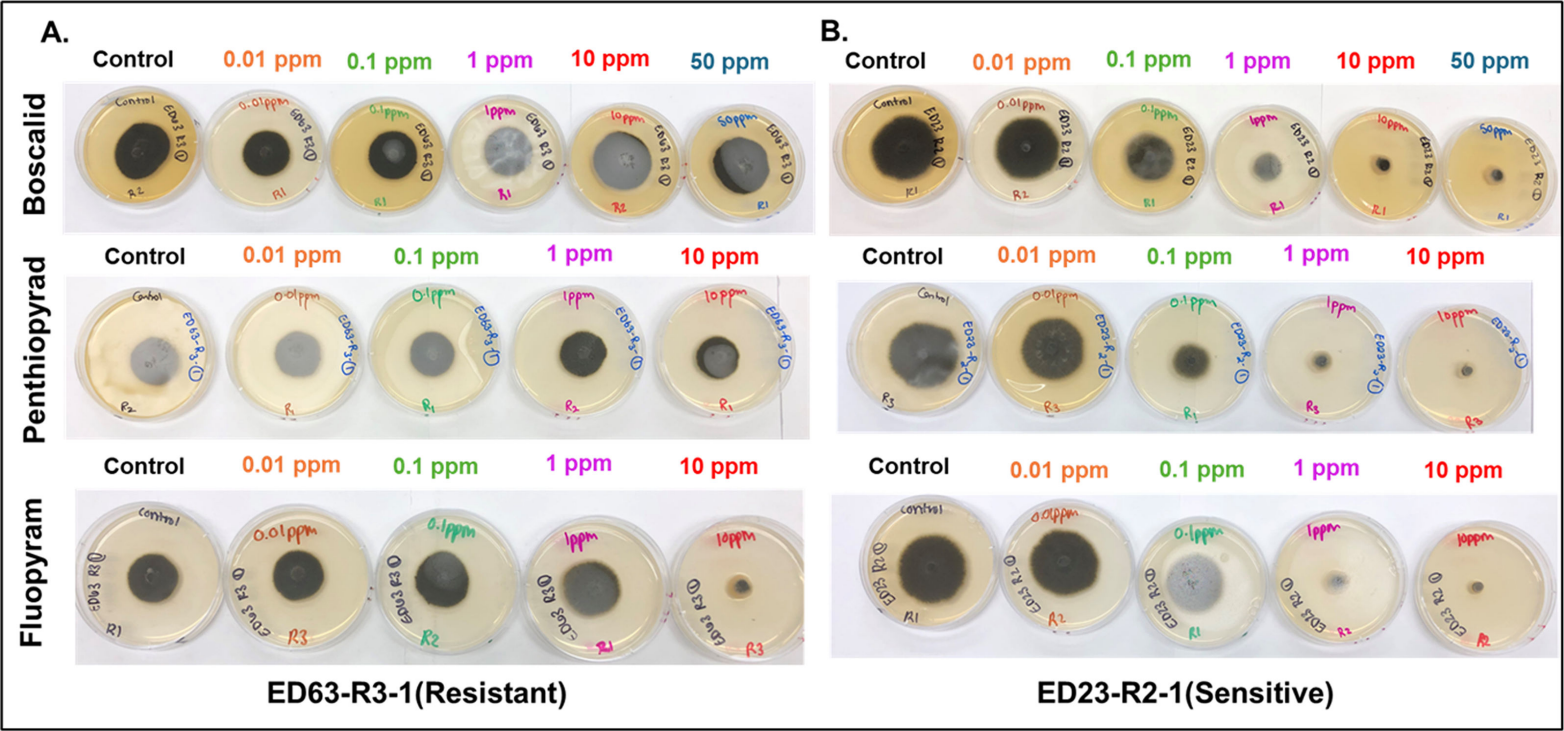
Radial growth assay of *Alternaria brassicicola* isolates (*n* = 58) from naturally infested commercial broccoli seedlots to three SDHI fungicides: boscalid, penthiopyrad, and fluopyram. Isolates were grown on both non-fungicide amended full-strength potato dextrose agar (PDA) and PDA amended with various concentrations of three fungicides (boscalid, penthiopyrad, and fluopyram). The concentrations used for boscalid were 0 ppm (control), 0.01 ppm, 0.1 ppm, 1 ppm, 10 ppm, and 50 ppm, while for penthiopyrad and fluopyram, the following concentrations were used: 0 ppm (control), 0.01 ppm, 0.1 ppm, 1 ppm, and 10 ppm. Panels depict representations of radial growth assay for a resistant isolate (ED63-R3-1; panel A) and a sensitive isolate (ED23-R2-1; panel B).

A set of 58 representative isolates selected from our previous study (13) was evaluated for sensitivity to three SDHI fungicides: boscalid, penthiopyrad, and fluopyram using a radial growth assay under *in vitro* conditions. EC_50_ values varied considerably among the tested fungicides (Fig. 1). A majority of the *A. brassicicola* isolates (*n* = 54/58) from naturally infested commercial broccoli seeds were resistant to boscalid and penthiopyrad, indicating resistance and a classic case of cross-resistance within the SDHI fungicides group. In the case of boscalid, no EC_50_ was calculated for 93% (*n* = 54) of the isolates since growth at the highest concentration was observed, while the rest of the isolates (*n* = 4) had a mean EC_50_ (1.32 ppm) ranging from 0.86 to 2.17 ppm (Fig. 1; Table 3). In the case of penthiopyrad, an EC_50_ value was not calculated for 93% (*n* = 54) of the isolates, while 7% (*n* = 4) had a mean EC_50_ value of 0.35 ppm, ranging from 0.22 to 0.61 ppm. The EC_50_ values for isolates varied significantly for the fungicide, fluopyram, with the values ranging from 0.45 to 5.23 ppm (mean EC_50_ = 1.95 ppm) (Table 3). Ninety-three percent of the isolates (*n* = 54) had EC_50_ values 60-fold greater than the most sensitive isolate (ED23-R2-1, 0.97 ppm) in the case of boscalid, while the same set of isolates had a 45-fold greater EC_50_ values than the most sensitive isolate (ED23-R2-1, 0.22) for penthiopyrad fungicide (Table 3, Fig. 2).

**TABLE 3:** Sensitivity of *Alternaria brassicicola* isolates (*n* = 58) from naturally infested commercial broccoli seedlots to three succinate dehydrogenase inhibitor fungicides, boscalid, penthiopyrad, and fluopyram, in a radial growth assay and mutations in the sdh genes (*sdh*B, *sdh*C, and *sdh*D)

Isolate number	*A. brassicicola* isolates^*a*^	Boscalid^*d*^		Penthiopyrad		Fluopyram		*sdh* gene mutations^*f*^			Detection of allele-specific *sdh*C mutation^*g*^
		EC_50_^*b*^ (ppm)	RF^*c*^	EC_50_ (ppm)	RF	EC_50_ (ppm)	RF	*sdh*B	*sdh*C	*sdh*D	
1	EC23-R3-3	>50.0	60	>10.0	45	5.23 a^*e*^	12	-	H134R	-	+
2	EC26-R1-1	>50.0	60	>10.0	45	1.78 b–k	4	-	H134R	-	+
3	EC2-R2-1	1.29	1	0.34	2	0.61 no	1	-	-	-	-
4	EC36-R1-2	>50.0	60	>10.0	45	4.05 ab	9	-	H134R	-	+
5	EC38-R2-2	>50.0	60	>10.0	45	2.48 a–h	6	-	H134R	-	+
6	EC42-R1-2	>50.0	60	>10.0	45	2.30 a–h	5	-	H134R	-	+
7	EC43-R2-2	0.87	1	0.24	1	0.75 k–o	2	-	-	-	-
8	EC4-R2-1	>50.0	60	>10.0	45	1.73 b–k	4	-	H134R	-	+
9	EC52-R3-1	>50.0	60	>10.0	45	2.14 a–i	5	-	H134R	-	+
10	EC53-R2-1	>50.0	60	>10.0	45	1.66 b–k	4	-	H134R	-	+
11	EC54-R1-2	>50.0	60	>10.0	45	1.74 b–k	4	-	H134R	-	+
12	EC55-R2-1	>50.0	60	>10.0	45	1.92 b–j	4	-	H134R	-	+
13	EC56-R3-2	>50.0	60	>10.0	45	2.18 a–i	5	-	H134R	-	+
14	EC58-R2-1	>50.0	60	>10.0	45	1.65 b–l	4	-	H134R	-	+
15	EC59-R1-1	>50.0	60	>10.0	45	2.92 a–f	7	-	H134R	-	+
16	EC60-R1-1	>50.0	60	>10.0	45	2.13 a–i	5	-	H134R	-	+
17	EC66-R3-1	>50.0	60	>10.0	45	2.1 a–i	5	-	H134R	-	+
18	EC69-R1-2	>50.0	60	>10.0	45	2.32 a–h	5	-	H134R	-	+
19	EC72-R2-1	>50.0	60	>10.0	45	1.84 b–k	4	-	H134R	-	+
20	EC74-R3-1	>50.0	60	>10.0	45	1.81 b–k	4	-	H134R	-	+
21	EC79-R1-1	>50.0	60	>10.0	45	1.61 b–m	4	-	H134R	-	+
22	EC84-R1-2	>50.0	60	>10.0	45	2.19 a–i	5	-	H134R	-	+
23	EC85-R3-2	>50.0	60	>10.0	45	2.74 a–g	6	-	H134R	-	+
24	EC87-R3-2	>50.0	60	>10.0	45	2.84 a–f	6	-	H134R	-	+
25	EC88-R1-1	2.17	2	0.61	3	0.77 j–o	2	-	-	-	-
26	EC89-R2-1	>50.0	60	>10.0	45	1.89 b–j	4	-	H134R	-	+
27	EC8-R2-1	>50.0	60	>10.0	45	1.41 e–n	3	-	H134R	-	+
28	EC91-R3-1	>50.0	60	>10.0	45	3.92 a–c	9	-	H134R	-	+
29	EC92-R2-1	>50.0	60	>10.0	45	1.55 d–m	3	-	H134R	-	+
30	EC94-R3-1	>50.0	60	>10.0	45	3.62 a–d	8	-	H134R	-	+
31	EC98-R2-1	>50.0	60	>10.0	45	3.31 a–e	7	-	H134R	-	+
32	ED100-R1-1	>50.0	60	>10.0	45	2.04 b–i	5	-	H134R	-	+
33	ED23-R2-1	0.97	1	0.22	1	0.45 o	1	-	-	-	-
34	ED32-R2-1	>50.0	60	>10.0	45	1.4 e–n	3	-	H134R	-	+
35	ED33-R3-1	>50.0	60	>10.0	45	1.64 b–l	4	-	H134R	-	+
36	ED35-R2-1	>50.0	60	>10.0	45	1.90 b–j	4	-	H134R	-	+
37	ED37-R3-2	>50.0	60	>10.0	45	1.32 e-n	3	-	H134R	-	+
38	ED38-R3-3	>50.0	60	>10.0	45	1.63 b–m	4	-	H134R	-	+
39	ED39-R3-1	>50.0	60	>10.0	45	2.03 b–i	5	-	H134R	-	+
40	ED3-R3-1	>50.0	60	>10.0	45	0.66 l–o	1	-	H134R	-	+
41	ED40-R1-1	>50.0	60	>10.0	45	2.73 a–g	6	-	H134R	-	+
42	ED44-R2-1	>50.0	60	>10.0	45	0.87 i–o	2	-	H134R	-	+
43	ED5-R1-1	>50.0	60	>10.0	45	1.59 c–m	4	-	H134R	-	+
44	ED61-R2-1	>50.0	60	>10.0	45	1.80 b–k	4	-	H134R	-	+
45	ED62-R2-4	>50.0	60	>10.0	45	0.65 m–o	1	-	H134R	-	+
46	ED63-R3-1	>50.0	60	>10.0	45	1.94 b–i	4	-	H134R	-	+
47	ED64-R1-1	>50.0	60	>10.0	45	1.79 b–k	4	-	H134R	-	+
48	ED68-R2-1	>50.0	60	>10.0	45	1.13 g–n	3	-	H134R	-	+
49	ED72-R3-1	>50.0	60	>10.0	45	1.29 f–n	3	-	H134R	-	+
50	ED73-R1-1	>50.0	60	>10.0	45	2.60 a–g	6	-	H134R	-	+
51	ED74-R2-1	>50.0	60	>10.0	45	1.65 b–l	4	-	H134R	-	+
52	ED80-R2-1	>50.0	60	>10.0	45	1.32 e–n	3	-	H134R	-	+
53	ED81-R1-2	>50.0	60	>10.0	45	1.98 b–i	4	-	H134R	-	+
54	ED84-R2-1	>50.0	60	>10.0	45	2.00 b–i	4	-	H134R	-	+
55	ED87-R3-1	>50.0	60	>10.0	45	0.94 h–o	2	-	H134R	-	+
56	ED91-R2-1	>50.0	60	>10.0	45	3.24 a–f	7	-	H134R	-	+
57	ED94-R1-1	>50.0	60	>10.0	45	2.05 b–i	5	-	H134R	-	+
58	ED9-R3-1	>50.0	60	>10.0	45	1.51 d–n	3	-	H134R	-	+

^*a*^ *A. brassicicola *isolates were isolated from commercial seedlots that were naturally infested from two broccoli cultivars (Cultivar 1 [EC] and Cultivar 2 [ED]).

^*b*^ EC_50_ is the effective fungicide concentration (in parts per million) at which the growth of an isolate is reduced by 50% compared with control.

^*c*^ The resistance factor (RF) was calculated by dividing the effective fungicide concentration (EC_50_) value of an isolate by the EC_50_ value of the sensitive isolate (ED23-R2-1).

^*d*^ Two independent radial growth experiments were conducted on a set of 58 representative *A. brassicicola* isolates in a completely randomized design with three replicates for each isolate. Means of EC_50_ are presented for boscalid and penthiopyrad as the isolates grew at the highest concentration screened (50 ppm and 10 ppm, respectively). While for fluopyram, analysis of variance was determined, and Tukey’s test was performed for mean separation.

^*e*^ Means in the column followed by the same letters are not significantly different according to Tukey’s honest significant difference (*P* < 0.05) test.

^*f*^ Mutation in the sdh genes in the target site corresponding to the SDHI fungicide resistance. The mutation in the sdhC gene (H134R; substitution of histidine by arginine at position 134 of sdhC gene) was observed in resistant isolates. - depicts no mutation in the *sdh* genes.

^*g*^ Allele-specific primer for screening H134R mutation in sdhC gene in the *A. brassicicola* isolates. + denotes the presence of H134R mutation, while - denotes the absence of mutation.

We observed that a set of isolates (*n* = 9) that had 100-fold reduced sensitivity than the most sensitive isolate to a QoI fungicide (azoxystrobin) as determined in our previous study (13), also had reduced sensitivity to boscalid (60-fold), penthiopyrad (45-fold), and fluopyram (2-7-fold), respectively (Table S1). The mean EC_50_ value of the sensitive isolate (ED23-R2-1) for azoxystrobin was <0.001 ppm, 0.86 ppm for boscalid, 0.24 ppm for penthiopyrad, and 0.75 ppm for fluopyram. A total of 15.5% (*n* = 9/58) of the isolates screened had reduced sensitivity to both QoI and SDHI fungicides. Although there was a difference in sensitivity to fluopyram for which isolates had varied EC_50_ values ranging from 1.73 ppm to 3.24 ppm, the EC_50_ values greater than 50 ppm (boscalid) and 10 ppm (penthiopyrad) were observed for these isolates against the other two SDHI fungicides (Table S1).

### Mutations in the *sdh* genes potentially confer resistance to SDHI fungicides in *A. brassicicola* isolated from naturally infested commercial broccoli seeds

The primers designed for the *sdh*B, *sdh*C, and *sdh*D subunits consistently amplified the expected regions across all tested isolates. Initial validation using four isolates (19-5 [sensitive to QoI; Georgia, USA]; EC43-R2-2 [sensitive to Boscalid; Georgia, USA]; ED63-R3-1 [resistant to Boscalid; Georgia, USA] and 23209 [resistant to Boscalid; New York, USA]) demonstrated successful amplification of the full-length genes with amplicon sizes matching those predicted (Table 1). Sequencing of these amplicons confirmed 98.7%–100% sequence coverage and 100% query coverage to the reference genes from our genomes of *A. brassicicola* strains. We also confirmed the sequence similarity (100%) with the Abra43 strain (Genome assembly: ASM279673v1, GeneBank Id: GCA_002796735.1) reference genome. No discrepancies or unexpected bands were observed during PCR amplification.

**Fig 3:**
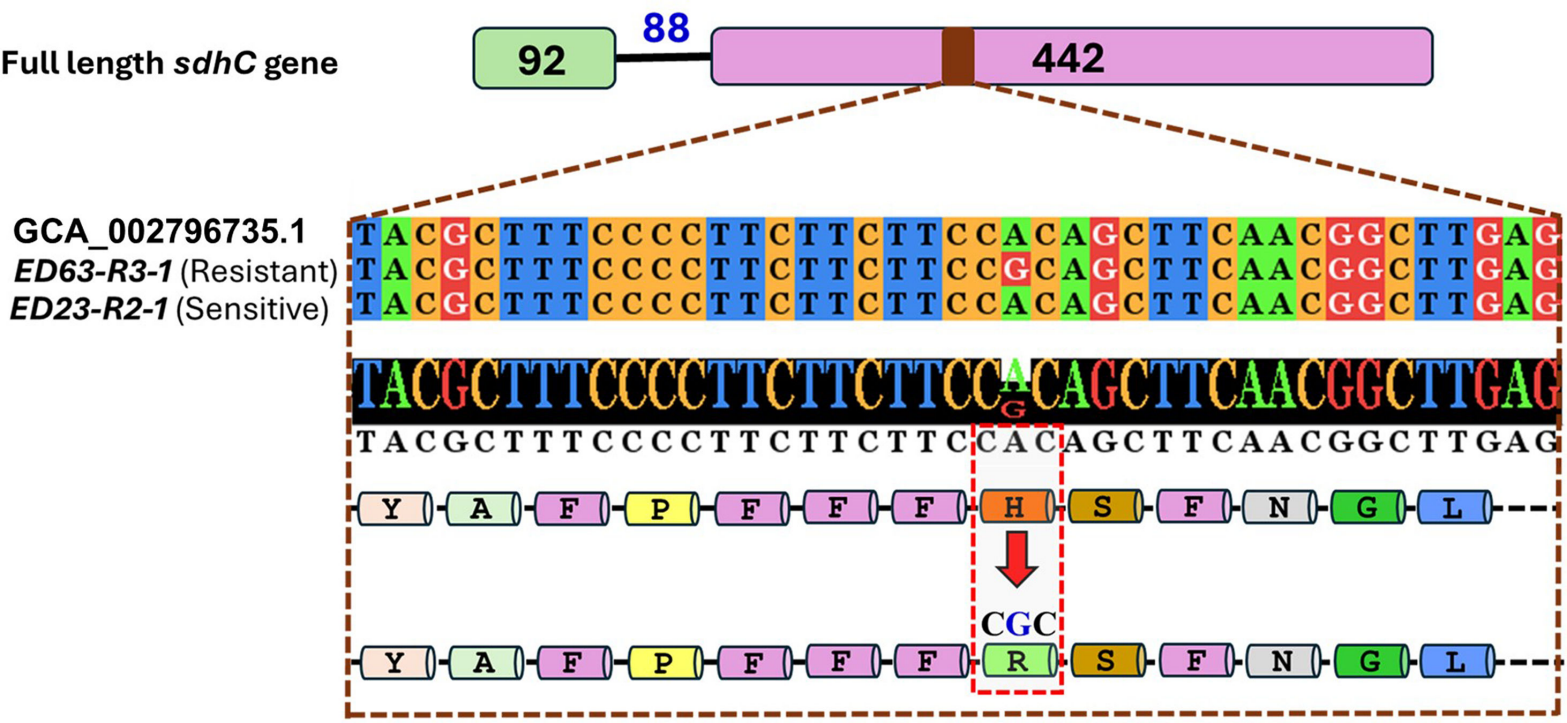
Comparison of a small region of the full-length reference *sdhC* gene of *A. brassicicola* with two isolates, highlighting a point mutation identified in the resistant isolate. This mutation, consistently observed across 54 resistant isolates but absent in four sensitive isolates, resulted in the substitution of histidine by arginine at position 134 of the *sdh*C gene.

Further testing of the primers on the remaining 54 isolates yielded amplicons of consistent sizes (*sdh*B: 1084 bp; *sdh*C: 963 bp; and *sdh*D: 977 bp) on agarose gels. Sanger sequencing revealed a single point mutation in the *sdh*C gene at the 134 amino acid position (H134R), resulting from an A/G nucleotide transition (Fig. 3). This mutation was exclusively present in all 54 boscalid- and penthiopyrad-resistant isolates (as determined by plating assay above), while the 4 boscalid- and penthiopyrad-sensitive isolates matched with the reference genome of *A. brassicicola*. No mutations were observed in the *sdh*B or *sdh*D genes based on the full-length sequences of both genes across any of the isolates.

### Allele-specific PCR assay for detection of SDHI (boscalid and penthiopyrad) resistance in *A. brassicicola*

To validate the H134R mutation in the *sdh*C gene, allele-specific primers were designed to distinguish between mutant- and wild-type alleles. PCR amplification using mutant-specific primers successfully detected the mutation in all 54 boscalid- and penthiopyrad-resistant isolates, as evidenced by distinct bands observed on agarose gels. By contrast, no amplification was observed in the four boscalid- and penthiopyrad-sensitive isolates, consistent with the absence of the mutation.

The allele-specific primers demonstrated high specificity that reliably distinguished the boscalid- and penthiopyrad-resistant isolates harboring the mutation from the boscalid- and penthiopyrad-sensitive isolates. This mutation, validated by Sanger sequencing and gel electrophoresis, offers a robust molecular marker for SDHI (boscalid and penthiopyrad) resistance in *A. brassicicola* that could facilitate future monitoring of fungicide resistance.

### Phylogenetic and genomic analyses of SDHI genes

**Fig 4:**
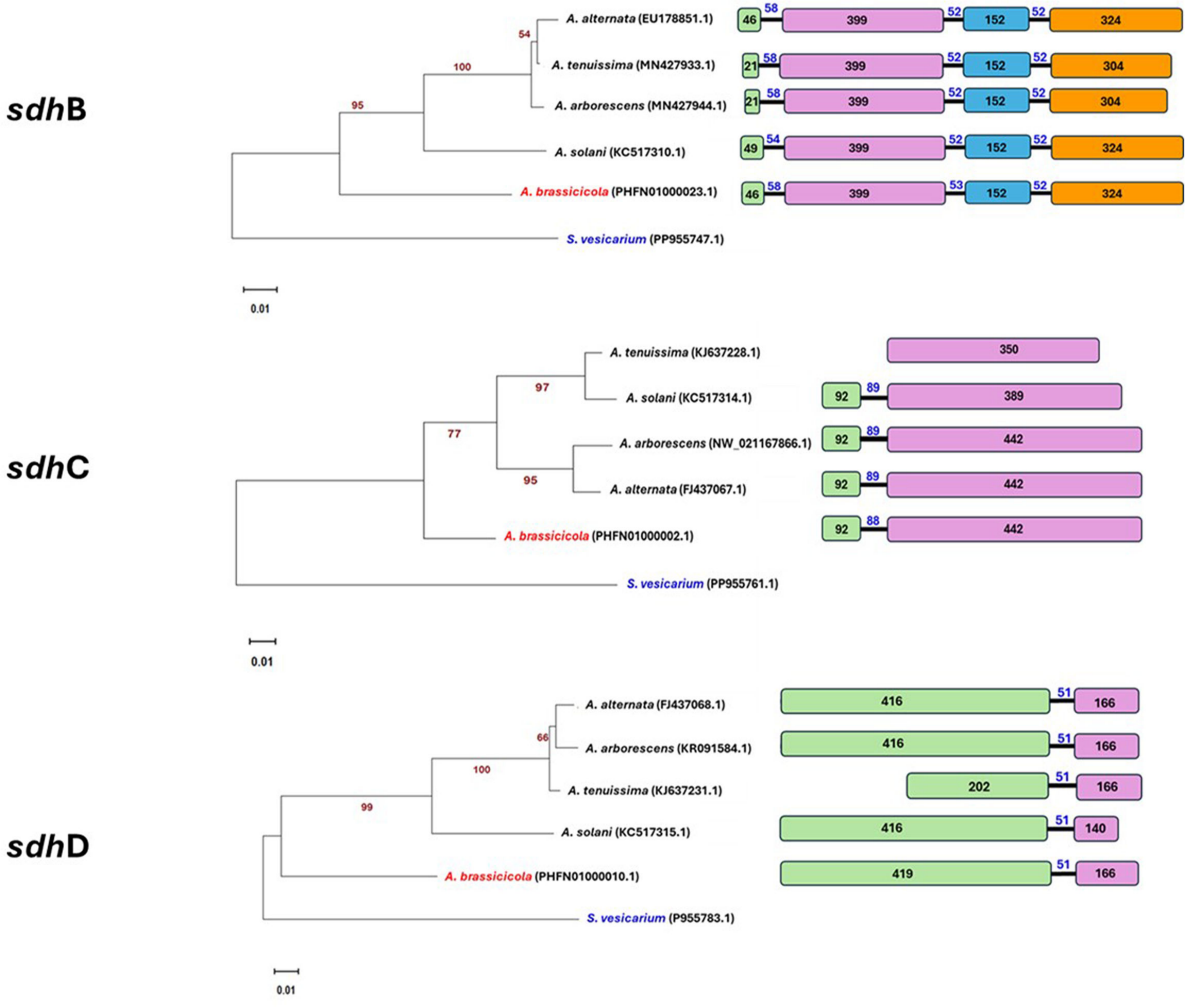
Comparison of structural organization of exons within three SDH subunits across multiple Al*ternaria* spp. isolates in the National Center for Biotechnology Information (NCBI). Variations in exon count and length across strains suggest evolutionary divergence, functional adaptation, or strain-specific traits. Each horizontal bar represents a distinct strain, with blocks denoting exons and connecting lines indicating introns. The evolutionary history was inferred using the Maximum Likelihood method and the Tamura-Nei model. The tree with the highest log likelihood for sdh-B (−2671.99), sdh-C (−1777.64), and sdh-D (−1779.14) is shown. The bootstrap consensus tree inferred from 500 replicates is taken to represent the evolutionary history of the taxa analyzed. The percentage of trees in which the associated taxa clustered together is shown next to the branches. Initial tree(s) for the heuristic search were obtained automatically by applying Neighbor-Join and BioNJ algorithms to a matrix of pairwise distances estimated using the Tamura-Nei model, and then selecting the topology with the superior log likelihood value. The tree is drawn to scale, with branch lengths measured in the number of substitutions per site. Evolutionary analyses were conducted in MEGA11.

Phylogenetic analysis of the SDHI subunits (*sdh*B, *sdh*C, and *sdh*D) across *A. brassicicola*, *A. solani*, *A. alternata*, *A. tenuissima*, and *A. arborescens* revealed distinct evolutionary relationships (Fig. 4). Maximum likelihood phylogenetic trees were constructed using the Tamura-Nei model, with the highest log likelihood values for *sdh*B (−2671.99), *sdh*C (−1777.64), and *sdh*D (−1779.14). Bootstrap analysis with 500 replicates provided strong support for the evolutionary relationships, with key branches displaying values exceeding 90% in most cases.

The phylogenetic trees demonstrated clear clustering of *A. brassicicola* with *A. solani* and *A. alternata*, reflecting close evolutionary relationships. However, the trees also revealed species-specific divergence patterns, particularly for the *sdh*C gene, which aligns with its functional importance and evolutionary variability.

Exon-intron structural analysis revealed conserved exon arrangements across *Alternaria* species, with minor variations in intron length and composition (Fig. 4). For example, while the exon count remained consistent across species, intron length in the *sdh*C subunit differed between *A. brassicicola* and *A. solani*, suggesting evolutionary adaptations specific to each species. This detailed comparison of exon-intron organization provides insights into the functional conservation and potential regulatory differences among SDHI genes in *Alternaria* species.

### Fitness of *A. brassicicola* from naturally infested commercial broccoli seeds with varying levels of fungicide sensitivity profiles

**Fig 5:**
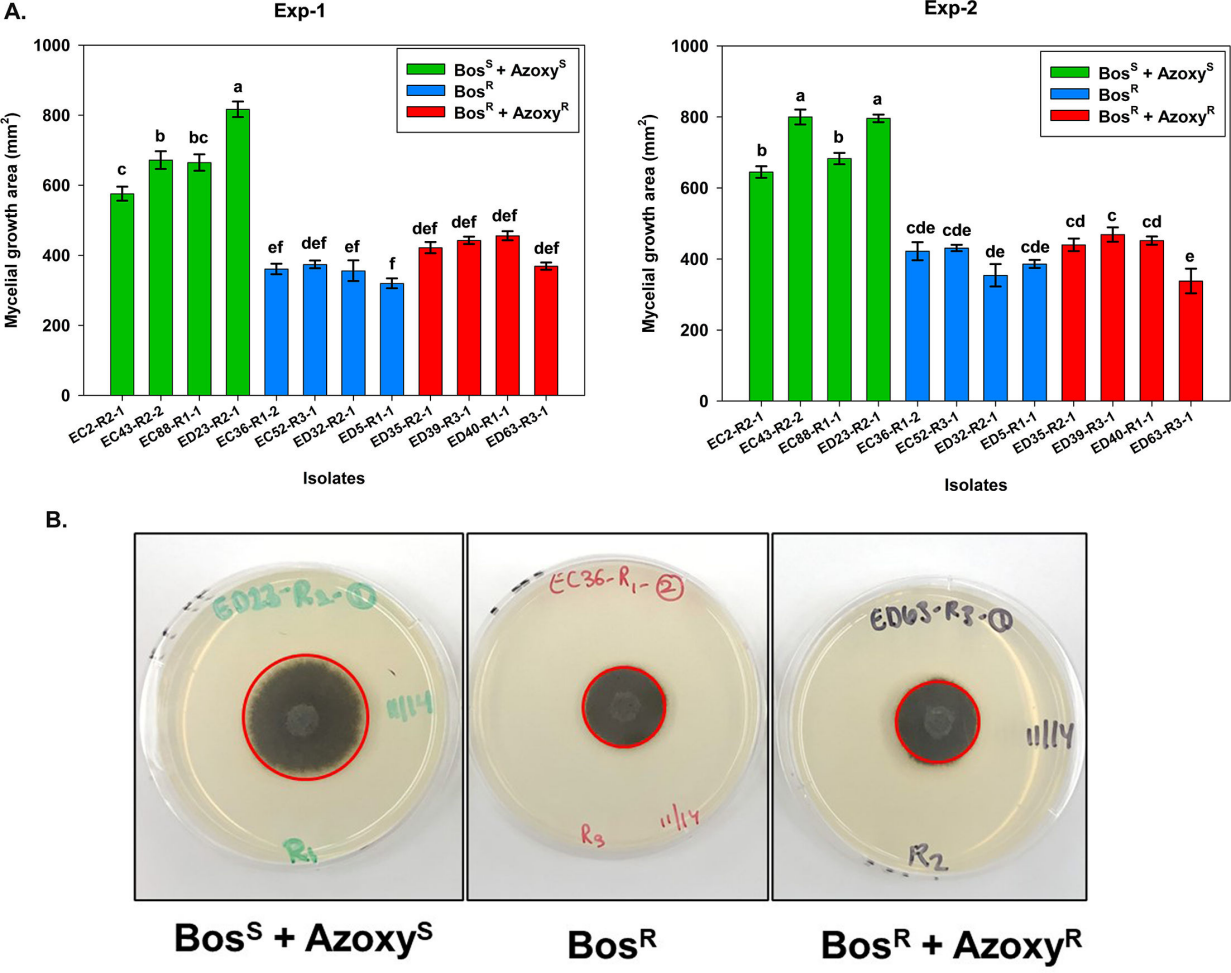
Pathogen fitness characteristics in terms of radial growth for *Alternaria brassicicola* isolates (*n* = 12) from naturally infested commercial broccoli seedlots with different levels of sensitivity profiles to azoxystrobin and three SDHI fungicides: boscalid, penthiopyrad, and fluopyram. Radial growth assay was determined for a set of isolates with varying sensitivity profiles to azoxystrobin and boscalid; set 1: isolates resistant to boscalid (Bos^R^, *n* = 4), set 2: isolates that are sensitive to both boscalid and azoxystrobin (Bos^S^ +Azoxy^S^, *n* = 4), and set 3: isolates that showed resistance to boscalid and less sensitive to azoxystrobin (Bos^R^ +Azoxy^R^, *n* = 4). Mean mycelial growth area (mm^2^) for each isolate belonging to each set was determined, and means were separated by Tukey’s honest significant difference (HSD) test after ascertaining their significance using ANOVA at *P* = 0.05. The panels show the following: (**A**) the mean mycelial growth area (mm^2^) of 12 isolates with varying sensitivity profiles to azoxystrobin and boscalid in two independent experiments (Exp-1 and Exp-2). The isolates in each category or set are color-coded; (**B**) the pictorial representation of mycelial growth (marked with red circle) of three isolates in each category or set; set 1: Bos^S^ +Azoxy^S^ (ED23-R2-1), set 2: Bos^R^ (EC36-R1-2), set 3: Bos^R^ +Azoxy^R^ (ED63-R3-1) on full-strength potato dextrose agar after 5 days of incubation.

Pathogen fitness characteristics of *A. brassicicola* with varying levels of sensitivity to azoxystrobin and three SDHI fungicides, boscalid, penthiopyrad, and fluopyram, were determined by evaluating mycelial growth and conidial germination. Three sets or categories of isolates were used to evaluate the pathogen fitness that included, set 1: isolates resistant to boscalid (Bos^R^, *n* = 4), set 2: isolates that were sensitive to both boscalid and azoxystrobin (Bos^S^ +Azoxy^S^, *n* = 4), and set 3: isolates that are resistant to boscalid and less sensitive to azoxystrobin (Bos^R^ +Azoxy^R^, *n* = 4). In case of mycelia growth, two independent experiments were conducted with four replicates of each isolate under *in vitro* conditions. Since there was significant interaction between the two experiments, both experiments were analyzed separately. For Exp-1, mean mycelial growth rates were 682.5 mm^2^, 352.7 mm^2^, and 422.3 mm^2^, for Bos^S^ + Azoxy^S^, Bos^R^, and Bos^R^ + Azoxy^R^, respectively. Isolates that are resistant to boscalid and less sensitive to azoxystrobin (Bos^R^ + Azoxy^R^) had significantly lower mycelial growth as compared to the isolates that were sensitive to these fungicides (*P* < 0.001) (Fig. 5A). However, the isolates that were resistant to either boscalid or resistant to both boscalid and less sensitive to azoxystrobin did not differ significantly in their mycelial growth from each other (Fig. 5A and B).

Similarly, for Exp-2, mycelial growth was significantly lower for the isolates that were resistant to boscalid (Bos^R^) and isolates that were resistant to boscalid and also less sensitive to azoxystrobin (Bos^R^ +Azoxy^R^). The isolates from these categories or phenotypes had significantly lower mycelial growth as compared to the isolates that were sensitive to both the fungicides (Bos^S^ +Azoxy^S^) (*P* < 0.001) (Fig. 5A). The mean mycelial growth rates observed for the isolates with phenotype, Bos^S^ +Azoxy^S^, Bos^R^, and Bos^R^ +Azoxy^R^ were 731.1 mm^2^, 398.3 mm^2^, and 424.8 mm^2^, respectively. However, the isolates that were resistant to boscalid alone or resistant to boscalid and less sensitive to azoxystrobin did not differ significantly in terms of their mycelial growth from each other (Fig. 5A and B).

However, for pathogen fitness as determined by assessing spore germination, we did not find any significant interaction between two independently replicated experiments; hence, the percent spore germination data were combined for both experiments and analyzed together (Fig. 4). Regardless of the fungicide sensitivity phenotypes, *A. brassicicola* isolates did not differ significantly in spore germination (*P* = 0.069). Isolates that were resistant to boscalid (Bos^R^) and resistant to boscalid and less sensitive to azoxystrobin (Bos^R^ +Azoxy^R^) had similar percent spore germination as that of isolates that were sensitive to both boscalid and azoxystrobin (Bos^S^ +Azoxy^S^) (Fig. S1).

## DISCUSSION

Our previous study categorically demonstrated that the isolates of *A. brassicicola* that were pathogenic on broccoli and less sensitive to a QoI fungicide (azoxystrobin) can be associated with commercial broccoli seeds (13). Apart from QoI fungicides, broccoli growers heavily rely on SDHI fungicides (boscalid, penthiopyrad, and fluopyram) for managing *A. brassicicola* (25, 26). Hence, it is imperative to assess the sensitivity of *A. brassicicola* isolates from naturally infested seeds against three commonly used SDHI fungicides in broccoli.

In this study, we evaluated *A. brassicicola* isolates from naturally infested commercial broccoli seedlots against three SDHI fungicides under *in vitro* conditions. Our findings provide the first molecular and phenotypic evidence that *A. brassicicola* from naturally infested commercial broccoli seeds in the United States exhibited cross-resistance to two SDHI fungicides (boscalid, penthiopyrad). The majority of isolates (93%) showed resistance to boscalid and penthiopyrad, with high EC₅₀ values (>50 ppm for boscalid and >10 ppm for penthiopyrad), suggesting cross-resistance to two SDHI fungicides in *A. brassicicola* isolates from seeds. This was further validated at the molecular level, where 93% of the resistant isolates harbored a point mutation (H134R) in the *sdh*C gene subunit, a well-documented marker of SDHI resistance in *Alternaria* spp. (16, 27). These observations may potentially explain regular occurrences of failure in managing *A. brassicicola* using boscalid and penthiopyrad under field conditions in Georgia, United States.

Interestingly, we observed variability in EC₅₀ values for fluopyram (ranging from 0.45 to 5.23 ppm), suggesting that sensitivity to this fungicide in *A. brassicicola*, which concurs with observations made in the field where reports of field insensitivity are yet to be reported. Differential sensitivity to boscalid and penthiopyrad versus fluopyram in *A. brassicicola* can be explained by their structural differences within the SDHI chemical class. Fluopyram belongs to the pyridinyl-ethyl-benzamide subclass, whereas boscalid belongs to the pyridine-carboxamides and penthiopyrad belongs to the pyrazole-carboxamides subclass. These structural variations affect fungicide binding to the succinate dehydrogenase enzyme, leading to differences in inhibitory efficiency. As a result, mutations in the SDH complex that confer resistance to one SDHI subclass may not always display cross-resistance to fluopyram.

Our findings further revealed that 15.5% of the *A. brassicicola* isolates that displayed a high EC_50_ value to azoxystrobin (13) also exhibited resistance to boscalid and penthiopyrad. These observations suggest that *A. brassicicola* isolates with reduced sensitivity and potentially resistant to two common FRAC groups of fungicides (QoI and SDHI) can occur in naturally infested broccoli seeds, a potential indication of the introduction of fungicide resistance via seeds. Since these isolates are also recovered from commercial broccoli seedlots, it is possible that *A. brassicicola* with reduced sensitivity to fungicides (QoI and SDHI) can be disseminated to regions where these fungicides have never been used in broccoli or in other brassica crops. Also, it may partly explain the sudden loss of sensitivity to both fungicides (SDHI [boscalid and penthiopyrad] and QoI [azoxystrobin]) in broccoli growing regions of the eastern United States.

SDHI resistance has been reported in multiple fungal species, including *A. alternata*, *A. solani*, *Botrytis cinerea*, and *Venturia inaequalis* (28, 29). Mutations in the SDH enzyme complex, particularly in *sdh*B, *sdh*C, and *sdh*D, induce structural changes that impair SDHI binding and confer resistance (22, 30–32). In our study, except for *sdh*C, we did not find mutations in any other *sdh* genes (*sdh*B and *sdh*D). The only mutation observed in *sdh*C was H134R in SDHI-resistant (boscalid and penthiopyrad) isolates. The H134R mutation identified in our study aligns with prior reports showing that homologous mutations in the *sdh*C gene confer resistance to multiple SDHI fungicides in *A. solani* (16, 22). In our study, we did not observe any other known mutations in the *sdh*C gene as reported in other *Alternaria* spp. (S135R, G79R) (23, 29, 33). The H134R mutation has been reported in other fungi as well, such as *Didymella tanaceti* (the causal agent of tan spot of pyrethrum [*Dalmatian pellitory*]), where resistance was observed against boscalid (30). In another fungal species, *Pyrenophora teres,* that causes net blotch in barley, multiple mutations in the *sdh*C gene, including H134R, were reported (31). Previous reports of mutations in several *sdh* genes were reported in *A. alternata* in almonds (33) and pistachio (29). In pistachio, *A. alternata* with several amino acid substitutions (*sdh*B: H277Y/R/L, P230 A/R/I/F,D, N235D/T/E/G; *sdh*C: H134R, S135R; *sdh*D: D123E, H133P) in boscalid- and fluopyram-resistant phenotypes were reported. In almonds, the most common mutation observed was H134R in *sdh*C, while other mutations were also depicted that included H277Y and H277L in *sdh*B, as well as G79R and S135R in *sdh*C. In addition, mutations like H277Y (*sdh*B) and S135R (*sdh*C) conferred high levels of resistance to boscalid and also resulted in reduced sensitivity to pyraziflumid, fluxapyroxad, and isofetamid (33).

To facilitate the rapid detection and monitoring of SDHI-resistant isolates, we developed allele-specific markers targeting the H134R mutation. This primer pair successfully distinguished SDHI-resistant isolates from the sensitive ones, demonstrating their utility for field monitoring and resistance surveillance. Similar molecular tools have been widely adopted for early detection of SDHI-resistant *Alternaria* isolates, improving disease management decisions and sustainable disease management (16, 27).

Phylogenetic analysis of the SDHI target genes across *Alternaria* species revealed strong clustering of *A. brassicicola* with *A. solani* and *A. alternata*, supporting their close evolutionary relationship (34). The conservation of exon-intron structures across *Alternaria* spp. suggests that mutations leading to fungicide resistance are likely constrained by evolutionary pressures, influencing the rate at which new mutations emerge (35). However, despite this evolutionary conservation, the observed differences in SDHI sensitivity among *Alternaria* species raise important questions about the role of species-specific genetic adaptations in fungicide resistance. It is not clear if variations in exon-intron architecture contribute to differential gene expression patterns that influence SDHI resistance levels. In addition, minor variations in intron lengths identified in our study could potentially be associated with alternative splicing events or regulatory modifications that impact SDH enzyme function. These regulatory elements could play a crucial role in determining resistance expression and stability over multiple growing seasons. Future research should explore whether these intron length variations correlate with differential resistance phenotypes across *Alternaria* species. Comparative transcriptomic and epigenetic studies could help determine whether resistance-related mutations are influenced by regulatory mechanisms beyond direct coding sequence changes. Moreover, if there are undiscovered compensatory mutations that mitigate the fitness costs of SDHI resistance, this would allow fungicide-resistant isolates to persist in field populations. Addressing these gaps in knowledge will provide a clearer picture of the evolutionary dynamics of fungicide resistance. Addressing these questions will not only provide deeper insight into the molecular evolution of fungicide resistance in *Alternaria* spp. but also improve predictive models for resistance development, aiding in the design of more sustainable disease management strategies. Integrating functional genomics with population genetics may further elucidate whether these genetic changes impact fitness trade-offs or adaptive advantages in resistant isolates.

Fungicide-resistant isolates can vary in their fitness ability, as it is crucial for predicting the durability of resistant populations (34). In our study, we assessed fitness by measuring mycelial growth and conidial germination rates across *A. brassicicola* isolates with different QoI and SDHI sensitivity profiles. We found that boscalid-resistant isolates exhibited significantly reduced mycelial growth compared to the isolates with other fungicide sensitivity profiles. However, we did not observe any significant differences in spore germination rates among different resistant isolates, suggesting that resistant isolates could potentially remain competitive in natural populations. However, further detailed studies on fitness cost in relation to spore production and germination may shed some light in the future. These findings are consistent with earlier research on SDHI resistance in *A. alternata*, where growth penalties were observed in SDHI-resistant isolates but not in QoI-resistant isolates (29). Moreover, our data suggest that despite potential fitness costs, SDHI-resistant isolates can persist and contribute to disease outbreaks if selection pressure remains high. This highlights the need for integrated resistance management strategies to limit the spread of resistant *A. brassicicola* isolates in commercial broccoli production.

The increasing prevalence of SDHI resistance emphasizes the urgency of sustainable fungicide resistance management. Studies suggest that resistance can be mitigated through fungicide rotations, mixtures, and the inclusion of fungicides with multi-site mode of action (28, 29). In *Venturia inaequalis*, modifying SDHI application rates was shown to reduce the survival of resistant isolates, suggesting that optimizing fungicide doses could delay the evolution of fungicide resistance (32). In addition, alternative control strategies, including biological control agents, have been proposed as sustainable options for managing diseases caused by *Alternaria* spp. (36). Integrating molecular diagnostics for monitoring the presence of fungicide resistance with traditional field scouting will be crucial for developing effective resistance management programs (27).

Overall, our results suggest that commercial broccoli seedlots can be naturally contaminated with pathogenic and aggressive *A. brassicicola* isolates with cross-resistance to two SDHI fungicides. This is also the first report of the occurrence of multiple fungicide resistance (QoI and SDHI) in *A. brassicicola* from naturally infested seeds. We report that the H134R mutation in the *sdh*C gene may potentially be related to resistance to boscalid and penthiopyrad. We also developed a PCR assay that can differentiate *A. brassicicola* isolates with H134R mutation, which can be used to detect and monitor potential SDHI resistance, particularly to boscalid and penthiopyrad. Based on these findings, it seems stringent management of *A. brassicicola* in broccoli seed production fields along with seed health testing is imperative to limit seed infection and potential dissemination of fungicide resistance isolates locally and globally.

## SUPPLEMENTAL MATERIAL

Supplemental material aem.01083-25-s0001.docx Tables S1 to S3; Fig. S1.

ASM does not own the copyrights to Supplemental Material that may be linked to, or accessed through, an article. The authors have granted ASM a non-exclusive, world-wide license to publish the Supplemental Material files. Please contact the corresponding author directly for reuse.
