# Supplementary material for: Cross-resistance in Alternaria brassicicola from naturally infested broccoli seeds against two succinate dehydrogenase inhibitor fungicides
Source: Appl Environ Microbiol. 2025 Sep 4;91(10):e01083-25. doi: 10.1128/aem.01083-25 (PMC12542640; doi:10.1128/aem.01083-25)
Supplement: Supplemental material — Tables S1 to S3; Fig. S1. [file aem.01083-25-s0001.docx]

**Supplementary Tables**

**Supplementary Table 1.** Fungicide sensitivity of *Alternaria brassicicola* isolates (*n* = 10) from naturally infested commercial broccoli seedlots to three succinate dehydrogenase inhibitor fungicides, boscalid, penthiopyrad and fluopyram and a quinone outside inhibitor fungicide, azoxystrobin.

|  | **Azoxystrobin^w^** | |  | **Boscalid** | |  | **Penthiopyrad** | |  | **Fluopyram** | |
| --- | --- | --- | --- | --- | --- | --- | --- | --- | --- | --- | --- |
| ***A. brassicicola* isolates^z^** | **EC_50_^y^** | **RF^x^** |  | **EC_50_** | **RF** |  | **EC_50_** | **RF** |  | **EC_50_** | **RF** |
| EC43-R2-2 | 0.05 | 481 |  | 0.86 | 1 |  | 0.24 | 1 |  | 0.75 | 2 |
| EC4-R2-1 | 0.02 | 151 |  | >50.0 | 60 |  | >10.0 | 45 |  | 1.73 | 4 |
| EC59-R1-1 | 0.02 | 165 |  | >50.0 | 60 |  | >10.0 | 45 |  | 2.92 | 7 |
| ED35-R2-1 | 0.03 | 259 |  | >50.0 | 60 |  | >10.0 | 45 |  | 1.90 | 4 |
| ED39-R3-1 | 0.10 | 971 |  | >50.0 | 60 |  | >10.0 | 45 |  | 2.03 | 5 |
| ED40-R1-1 | 0.02 | 165 |  | >50.0 | 60 |  | >10.0 | 45 |  | 2.73 | 6 |
| ED63-R3-1 | 0.03 | 325 |  | >50.0 | 60 |  | >10.0 | 45 |  | 1.94 | 4 |
| ED84-R2-1 | 0.33 | 3332 |  | >50.0 | 60 |  | >10.0 | 45 |  | 2.00 | 4 |
| ED91-R2-1 | 0.04 | 363 |  | >50.0 | 60 |  | >10.0 | 45 |  | 3.24 | 7 |
| ED94-R1-1 | 0.02 | 158 |  | >50.0 | 60 |  | >10.0 | 45 |  | 2.05 | 5 |

^z^*A. brassicicola* isolates were isolated from commercial seedlots that were naturally infested from two broccoli cultivars (Cultivar 1 [EC] and Cultivar 2 [ED])

^y^EC_50_ is the effective fungicide concentration (in parts per million) at which the growth of an isolate is reduced by 50% compared with control.

^x^The resistance factor (RF) was calculated by dividing the effective fungicide concentration (EC_50_) value of an isolate by the EC_50_ value of the sensitive isolate (ED23-R2-1).

^w^ EC_50_ values of the isolates were taken from our previously conducted azoxystrobin sensitivity test using a spore germination assay (Kaur and Dutta., 2024).

**Supplementary Table 2.** *Alternaria brassicicola* isolates used in genome sequencing and downstream analysis for the development of succinate dehydrogenase inhibitor (SDHI) gene primers.

| **Collectors ID** | **State** | **Year** | **Host** | **Other info** |
| --- | --- | --- | --- | --- |
| 19-5 | GA | 2019 | Broccoli | Less sensitive isolate in Nieto-Lopez et al. 2023 |
| 18062 | NY | 2018 | Broccoli |  |
| F6A2 | GA | 2020 | Broccoli | Isolate collected from Tattnall County |
| F6A9 | GA | 2020 | Broccoli | Isolate collected from Tattnall County |

**Supplementary Table 3.** Accession numbers of *Alternaria* spp. used for the development of succinate dehydrogenase inhibitor (SDHI) gene primers for *A. brassicicola.*

| **SDHI Subunit** | **Species** | **Accession number** | **Reference** | **Isolate ID** |
| --- | --- | --- | --- | --- |
| *sdh*B | *A. alternata* | EU178851 | Avenot et al. 2009 | AaY16 |
| *sdh*C | *A. alternata* | FJ437067 | Avenot et al. 2008 |  |
| *sdh*D | *A. alternata* | FJ437068 | Avenot et al. 2008 |  |
| *sdh*B | *A. solani* | KC517310 | Mallik et al 2013 | 1178-W1 |
| *sdh*C | *A. solani* | KC517314 | Mallik et al 2013 | 1179-13 |
| *sdh*D | *A. solani* | KC517315 | Mallik et al 2013 | 1178-W1 |

**Supplementary Figures**

**Supplementary Fig. 1** Pathogen fitness characteristics in terms of spore germination for *Alternaria brassicicola* isolates (*n* = 12) from naturally infested commercial broccoli seedlots with different levels of sensitivity profiles to azoxystrobin and and three succinate dehydrogenase inhibitor fungicides (SDHI) fungicides, boscalid, penthiopyrad and fluopyram. Spore germination was determined for a set of isolates with varying sensitivity profiles to azoxystrobin and boscalid; set 1: isolates resistant to boscalid (Bos^R^, *n*=4), set 2: isolates that are sensitive to both boscalid and azoxystrobin (Bos^S^ + Azoxy^S^, *n*=4), and set 3: isolates that showed resistance to boscalid and less sensitive to azoxystrobin (Bos^R^ + Azoxy^R^ , *n*=4). Two independent experiments were conducted with four replicates in a completely randomized design. The conidium was considered germinated if the length of the germ tube was bigger than the size of the conidia. Mean spore gemination (%) for each isolate belonging to each set were determined and letters on the boxplots followed by the same letters are not significantly different according to Tukey’s honest significant difference (*P* < 0.05) test.


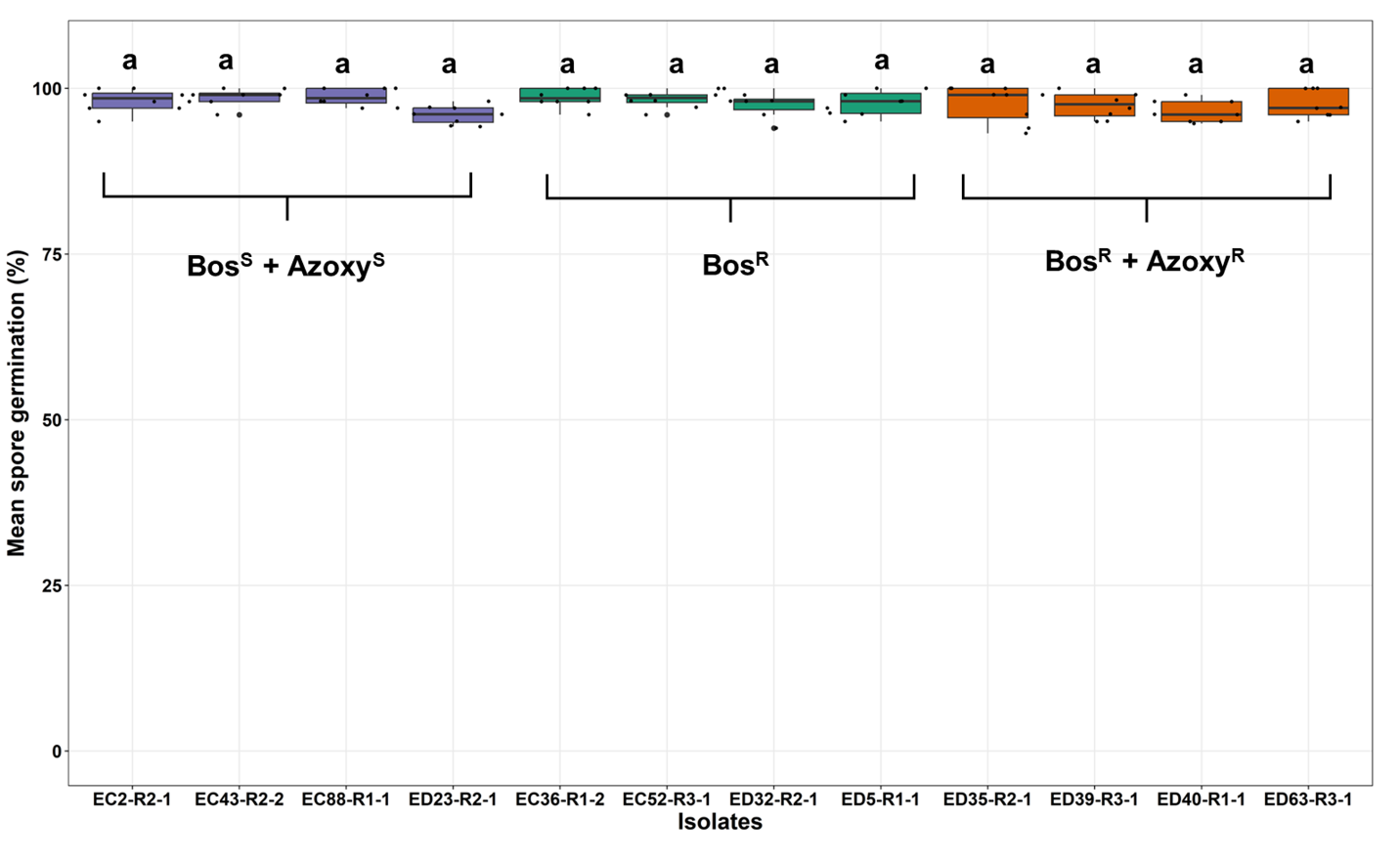


Reference:

1. Kaur N, Dutta B. 2024. Aggressive *Alternaria brassicicola* with reduced fungicide sensitivity can be associated with naturally infested broccoli seeds. *Plant Dis* 108:2154-2161.
